# Modeling Outbreak Prediction and the Impact of Emergency Vaccination on the 2024–2025 Chikungunya Outbreak in La Réunion

**DOI:** 10.3390/vaccines13121181

**Published:** 2025-11-21

**Authors:** Martijn Boer, Gerard Timmy Vondeling, Eric Plennevaux, Adrianne Marije de Roo

**Affiliations:** 1 Asc Academics B.V., 9725 AK Groningen, The Netherlands; 2 Valneva Austria GmbH, 1030 Vienna, Austria; 3 Valneva France SAS, 69002 Lyon, France; 4 Unit of Global Health, Department of Health Sciences, University Medical Centre Groningen, 9713GZ Groningen, The Netherlands

**Keywords:** chikungunya, outbreak, mosquito-borne disease, La Réunion, vaccination, *Aedes*, public health

## Abstract

Background/Objectives: As of April 2025, La Réunion is facing a second major chikungunya virus (CHIKV) outbreak, following the 2005–2006 epidemic that infected nearly one-third of the population. IXCHIQ^®^, a live-attenuated, single-dose vaccine, offers an opportunity for targeted immunization to complement vector control efforts. Using surveillance data up to 23 February 2025 (week 7), we estimated the potential scale of the 2024–2025 chikungunya outbreak in La Réunion and how much of the burden could have been averted by an emergency vaccination campaign at different detection thresholds. Methods: A stochastic SEIR–SEI host–vector model was calibrated to weekly case counts (weeks 46/2024–7/2025). We projected the epidemic under three vaccination-trigger scenarios (≥100, ≥3000, ≥40,000 detected cases) and two incremental vector-control assumptions (10% and 20% reductions in biting rate). Several mosquito-related parameters—extrinsic incubation period, offspring number, and mortality rate—were temperature-dependent, based on daily temperatures in La Réunion. Vaccination was applied homogeneously, using a 14.5% coverage to reflect the proportion of the population targeted in the initial public health recommendation. Results: Our findings indicate that without vaccination, up to 27.5% of the population could become infected. If vaccination would begin after 100 detected cases, 75% of infections could be prevented. Delaying until 3000 or 40,000 cases reduced effectiveness to 41% and 11%, respectively. Conclusions: Our results show that timely emergency vaccination can substantially reduce outbreak size. This underscores the importance of preparedness and rapid response by public health authorities in high-risk regions.

## 1. Introduction

From February 2005 to August 2006, the island of La Réunion was struck by a large-scale outbreak of chikungunya virus (CHIKV). Post-outbreak reports have estimated that approximately 300,000 people (or 38.2% of the population) were infected with CHIKV, with most infections being symptomatic and characterized by high fever, arthralgia, myalgia, headache, asthenia, and rash [1]. The outbreak led to substantial morbidity in the local population, including long-lasting symptoms and excess mortality [2,3]. Besides these health consequences, the outbreak also resulted in a significant economic burden, as total medical costs alone were estimated to amount to €43.9 million [4]. It was later calculated that the outbreak had a reproductive number of 3.7 and an estimated attack rate of 73%. However, the actual percentage of the population that was infected was 38.2%, which is significantly lower than the attack rate [5]. Therefore, researchers concluded that a resurgence of CHIKV on the island is very likely [1]. This prediction became reality in August 2024, when the first autochthonous case of CHIKV in ten years was reported in La Réunion [6]. After a couple of months, the number of cases increased sharply, as the outbreak spread into more areas. As of 23 February (week seven) of 2025, 695 new infections were reported, bringing the cumulative total to approximately 1745 infections. At that point, CHIKV cases had been detected in 22 out of 24 municipalities, with the highest number of cases occurring in Le Tampon and L’Étang-Salé [7].

A significant difference from the 2005–2006 outbreak is that a preventive vaccine is now available and approved in Europe. On 31 May 2024, the European Medicines Agency (EMA) approved the first chikungunya vaccine, IXCHIQ^®^ [8]. On 31 January 2025, another vaccine, Vimkunya^®^, received market authorization in the EU [9]. IXCHIQ^®^ is a single-dose, live-attenuated vaccine licensed for use in individuals aged 12 and older. Vimkunya^®^ is a single-dose, virus-like particle (VLP) vaccine licensed for individuals aged 12 and older. Both vaccines were approved based on immunological data and have been shown to induce strong and lasting immune responses, with seroprotective rates of 98.9% [10] and 97.8% [11] for IXCHIQ^®^ and Vimkunya^®^, respectively, in their phase 3 trials. Emergency vaccination against CHIKV has the potential to reduce the impact of any outbreak by lowering susceptibility to infection [12]. In La Réunion, a combined strategy of vaccination and vector control measures could be implemented to mitigate the impact of the outbreak by preventing it from spreading further [13]. To date, no research has been conducted about the impact of an emergency vaccination program for CHIKV in combination with vector control measures. Despite significant efforts on increased vector control, CHIKV cases kept increasing exponentially.

This study aims to assess the potential size of the CHIKV outbreak in La Réunion and assess the impact of an emergency vaccination program with IXCHIQ^®^. We calibrated the model to the real-life outbreak size data available at the time of its development to ensure an accurate representation of the early phase of the outbreak. This also improves the reliability of outbreak predictions by incorporating real-world outbreak dynamics into the model predictions. The model projects that the current epidemic trajectory could lead to a substantial outbreak, and an emergency vaccination program might prevent up to 75% of cases. Although the model is numerically tuned to the 2024–2025 La Réunion resurgence, its broader purpose is to demonstrate how different vaccination-trigger thresholds and vector-control gains translate into epidemic outcomes, thereby providing a transferable framework that other at-risk regions can adapt-well before the first local case is detected.

Because no approved vaccine was available during the 2005–2006 outbreak, public-health decisions relied mainly on vector control. The present resurgence, therefore, offers a natural experiment: if calibrated models can forecast the epidemic curve early on, they could guide the timing threshold for vaccine deployment during future outbreaks, when every week of delay may translate into tens of thousands of preventable cases.

## 2. Materials and Methods

### 2.1. Model Structure

**Figure 1:**
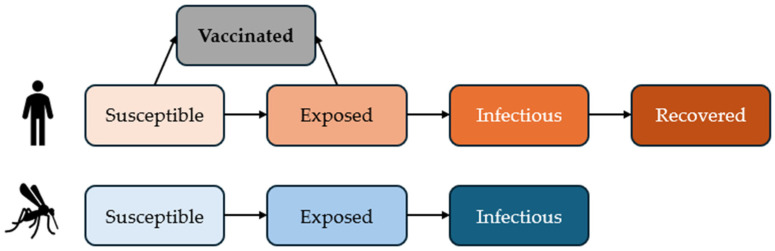
Overview of the SEIR-SEI model structure with an additional health state for vaccinated people.

We constructed a stochastic vector–host model to simulate the dynamics of the current CHIKV outbreak in La Réunion. The human host population was divided into Susceptible ($S_{h}$), Exposed ($E_{h}$), Infected ($I_{h}$), and Recovered ($R_{h}$) compartments, and the mosquito vector population in Susceptible ($S_{m}$), Exposed ($E_{m}$), and Infectious ($I_{m}$) compartments (SEIR-SEI model). In our model, a human was considered susceptible if they had neither received vaccination nor acquired natural immunity from the previous outbreak, approximately 20 years earlier. For mosquitoes, all individuals were assumed to be susceptible at the start of the model, as their short lifespan precludes the possibility of residual immunity from prior outbreaks. At timepoint zero (t = 0), approximately 800,000 susceptible mosquitoes were introduced into the model population. A vaccinated health state was added to the human compartment to simulate the emergency vaccination program scenario (Figure 1). Both susceptible and exposed humans could be vaccinated.

For both people and mosquitoes, the transitions between health states were determined based on ordinary differential equations (ODEs) [14]. A person could transition from the susceptible to the exposed state when bitten by a CHIKV-infected mosquito. Whether or not a person became exposed depends on the biting rate of mosquitoes (a), the human susceptibility to infection (b), the number of susceptible humans ($S_{h}$), the number of infected mosquitoes ($I_{m}$), and the total human population size ($N_{h}$). In the exposed state, an exposed person ($E_{h}$) has contracted the disease but is not yet infectious. The transition from an exposed to an infected human ($I_{h}$) corresponds to the inverse of the intrinsic incubation period ($\omega_{h}$). When individuals were no longer infectious, they moved to the recovered human state ($R_{h}$), which was determined by the recovery rate (σ). Given the short time horizon of the model simulations, human birth and mortality rates were not considered.

The human compartment of the model consists of the following ODEs:

(1) $$\frac{dS_{h}}{dt}=-ab\frac{S_{h}I_{m}}{N_{h}}$$

(2) $$\frac{dE_{h}}{dt}=ab\frac{S_{h}I_{m}}{N_{h}}-\omega_{h}E_{h}$$

(3) $$\frac{dI_{h}}{dt}=\omega_{h}E_{h}-\sigma I_{h}$$

(4) $$\frac{dR_{h}}{dt}=\sigma I_{h}$$

A mosquito transitioned from susceptible to exposed through contact with a CHIKV-infected human. Therefore, whether a mosquito is exposed depends on the biting rate of mosquitoes (a), the mosquito susceptibility to infection (c), the number of susceptible mosquitoes ($S_{m}$), the number of infected humans ($I_{h}$), and the total human population size ($N_{h}$). Similarly to the human compartment, exposed mosquitoes ($E_{m}$) could not transmit the disease. Mosquitoes could transmit CHIKV when they were infectious ($I_{m}$), so when the virus has incubated within their body. Therefore, the transition from exposed to infected corresponds to the inverse of the extrinsic incubation period ($\omega_{m}$). The size of the mosquito population is determined by the birth rate (δ) and mortality rate (μ).

The mosquito compartment of the model consists of the following ODEs:

(5) $$\frac{dS_{m}}{dt}=\delta N_{m}-ac\frac{S_{m}I_{h}}{N_{h}}-\mu S_{m}$$

(6) $$\frac{dE_{m}}{dt}=ac\frac{S_{m}I_{h}}{N_{h}}-\left(\mu +\omega_{m}\right)E_{m}$$

(7) $$\frac{dI_{m}}{dt}=\omega_{m}E_{m}-\mu I_{m}$$

At each time step within the simulation, the outcomes of these ODEs were calculated. Each outcome reflected the expected value for that specific transition rate at that time step. These expected values were used as inputs for a Poisson function. Using random number generation for probability values, we were able to obtain discrete values at each time step. We used 50 different random seeds for the random number generation, ensuring the reproducibility of results. Opting for this stochastic approach with discrete values allowed us to account for the inherent randomness of virus outbreaks. Each random seed yields different results, representing the various trajectories that the outbreak could potentially take.

### 2.2. Model Inputs

An overview of the inputs used in our model is presented in Table 1. Some mosquito-related parameters were temperature-dependent and are discussed in more detail later in this section.

Based on expert opinion and a report from the French National Authority for Health (HAS), we estimated the natural immunity due to the previous outbreak to be around 20% [5]. The exact vector–host ratio (VHR) in La Réunion is unknown, as it is impossible to calculate. Therefore, we assumed the VHR to be 2 and corrected this indirectly by calibrating the biting rate of the mosquitoes so that the model outcomes were closely aligned with real-life epidemiological data. Prior to week 46 of 2024, the number of infections fluctuated unpredictably from week to week, making it difficult to reliably calibrate the model. This irregularity is likely attributable to inherent randomness, not explanatory variables. Consequently, 17 November was selected as the starting point, since infections began to exhibit a consistent upward trend from that date onward.

Model calibration implicitly accounted for ongoing vector control activities. By calibrating the model to the ongoing outbreak in La Réunion, the mosquito biting rate was adjusted to reflect local outbreak dynamics. Any interventions undertaken in La Réunion during the calibration period that have an impact on the outbreak size—whether public measures such as using mosquito nets and emptying potential breeding sites or government actions like pesticide use—were incorporated in the model through their effect on the calibrated biting rate. However, vector control activities are likely to be strengthened as the outbreak progresses. Since the impact of these countermeasures is uncertain, we selected two possible values: 10% and 20%. This was implemented by lowering the biting rate of the mosquitoes.

Several mosquito-related parameters in the model were derived from daily temperature data for La Réunion. Using fixed values for these parameters would fail to capture seasonal temperature variations that influence critical factors such as incubation rate, birth rate, and mortality rate. The extrinsic incubation period is the time it takes the virus to develop inside the mosquito. This parameter is expressed by Equation (8) [18], with T being the daily temperature in La Réunion per time step. The offspring number provides the mean number of viable offspring produced by one female mosquito over her entire lifespan [19]. The effect of temperature on the offspring number is expressed by the polynomial that can be found in Equation (9), based on an article from Yang et al. [19]. Lastly, the effect of temperature on the mortality rate of mosquitoes is expressed by Equation (10) [19].

Equation (8). Intrinsic incubation rate.

(8) 4+exp(5.15−0.123T)

Equation (9). Offspring number.

(9) $$-341.8585+108.7373T-12.3447T^{2}+0.6367451T^{3}-0.0149469T^{4}+0.0001294166T^{5}$$

Equation (10). Mortality rate

(10) $$0.8692-0.1590T+0.01116T^{2}-0.0003408T^{3}+0.000003809T^{4}$$

### 2.3. Model Calibration

All model parameters were estimated using surveillance data reported through week 7 (ending 23 February 2025), the latest data available at the time of model building. The mosquito biting rate was adjusted weekly to align the model’s predicted infection numbers with observed cases. Calibration was first performed using a single random seed, after which simulations were run across all seeds. This approach allowed us to identify the seed that produced median results, which was then used for the final calibration.

For future projections, we assumed that the average trend in biting rate observed over the last three known weeks (weeks 5, 6, and 7) would continue until the outbreak reaches its anticipated peak. Based on expert opinion, this peak was expected in mid-May. Following the peak, we assumed a gradual decline in the biting rate, leading to the end of the outbreak by July. We assumed the end of the outbreak to be in July based on historical data from the previous outbreak. Model validity was checked by comparing hindcasts for weeks 8–13 to subsequently published surveillance totals (20,242 cases by 23 March 2025), which fell within the middle 50% of our predictive interval (see Section 4).

### 2.4. Vaccination Strategy

The vaccination scenario reflected the proportion of the population targeted in the initial public health recommendation, including individuals aged 65 years and older and individuals aged 18–64 years with comorbidities, as these groups were considered at higher risk of developing severe disease outcomes. This targeted approach was reflected in our assumed vaccine coverage rates, which did not include individuals aged 0–17 years or adults aged 18–64 years without comorbidities. It is essential to note that this vaccination campaign occurred prior to the emergence of real-world safety data, which led to a warning and precaution regarding use in the elderly population with underlying comorbidities. Therefore, future use should align with the latest label and recommendations for use.

Within these subgroups, we assumed that 50% of people would recall having developed natural immunity as a result of the previous outbreak. For this group, vaccination would not provide additional benefits, and therefore, they do not require vaccination. Furthermore, we assumed a 50% vaccination acceptance rate. See Table 2 for the expected numbers per age group for the targeted vaccination program.

As the core structure of this model does not account for age groups, we did not dynamically incorporate age-targeted vaccination strategies. Instead, we calculated the overall proportion of the population that would be vaccinated in this scenario, which amounted to 14.5%. Consequently, this value was used as the vaccination coverage rate in the simulations.

Additionally, we simulated different scenarios for the timing of the vaccination campaign initiations. The vaccination program is assumed to commence after a specific number of cases have been detected. We simulated three scenarios, representing intervention after 100, 3000, and 40,000 cases. When the vaccination program was initiated, we assumed that the coverage rate increased linearly over a 30-day period until the program reached its intended coverage of 14.5%. People who get vaccinated are not immediately protected against CHIKV, as it takes approximately 14 days for the vaccination to provide immunity [21]. These three different scenarios were analyzed in combination with an additional 10% or 20% vector control effectiveness, leading to a total of six scenarios.

## 3. Results

### 3.1. Outcomes of Model Calibration

At the time of model development, the latest known epidemiological data point of the real-life outbreak was the number of cumulative infections at week seven in 2025. At this time, the outbreak resulted in 1745 infections. After calibrating the model, the outcomes of each simulation, generated from the 50 random seeds, were compared to the observed 1745 infections to evaluate which simulations produced realistic results that followed the trajectory of the real-life outbreak. Simulations that deviated less than 15% from the observed number of infections—i.e., all runs that predicted between 1483 and 2007 infections at week seven—were considered similar to the real-life outbreak. A total of seven simulations yielded epidemic trajectories that closely resembled the trajectory of the real-life outbreak, with the number of infections ranging from 1706 to 1975. These seven simulations were selected for in-depth analysis. Simulations that led to runs diverging by more than 15% from this data point were excluded from our results analysis, as these outbreaks were deemed unrealistic and either too small or too large. In total, 17 simulations resulted in outbreak sizes that were considered unrealistically small, whereas 26 simulated outbreaks were considered unrealistically large.

During weeks 8–13 (not used for calibration), the model’s median forecast deviated <15% from observed case counts, indicating good short-range predictive accuracy.

### 3.2. Chikungunya Outbreak Size Predictions

Our model predicts that the current epidemic trajectory could lead to a substantial outbreak, infecting around 246,500 people in La Réunion (Table 3 and Table 4). This prediction is based on the scenario with an additional 10% vector control effectiveness. Across the seven random seeds that produced results in line with the current outbreak, the predicted total number of infections for the scenario with an additional 10% vector control effectiveness ranged from 188,926 to 298,861 (Table 3). When assuming a 20% increase in vector control effectiveness, the total outbreak size was approximately 108,000 people (Table 3 and Table 4). Across the seven random seeds, the results ranged from 72,065 to 147,514 (Table 3).

### 3.3. The Effect of Vaccination on the Outbreak Size

**Figure 2:**
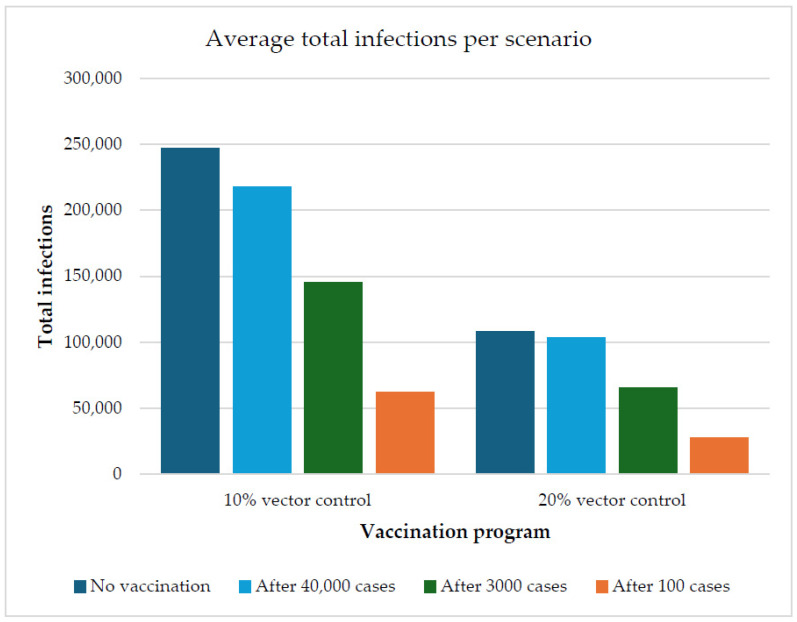
Visual representation of the average chikungunya outbreak size per scenario for the seven runs. Shown are the different vaccination strategies, with vaccination starting after 100 cases, 3000 cases, 40,000 cases, and no vaccination, for different vector control scenarios.

An emergency vaccination program with a coverage rate of 14.5% was evaluated at different initiation moments (i.e., after 100 cases, 3000 cases, or 40,000 cases). With initiation after 100 cases, the vaccination program was able to reduce the total number of infections by an average of 75.1% with an additional 10% vector control effectiveness and by an average of 74.7% with an additional 20% vector control effectiveness. The total number of infections in the vaccination scenario ranged from 44,018 to 90,726 and from 15,357 to 43,718, respectively (Table 4 and Figure 2).

When the vaccination program was initiated after 3000 cases, it reduced the total number of infections by an average of 41.4% in the scenario with an additional 10% in vector control effectiveness and 39.6% in the scenario with an additional 20% in vector control effectiveness. The total number of infections in this vaccination scenario dropped, ranging from 110,575 to 178,032 and 44,680 to 88,059, respectively (Table 4 and Figure 2).

When the vaccination program was initiated after 40,000 cases, it was able to reduce the total number of infections by an average of 11.4% in the scenario with an additional 10% increase in vector control effectiveness, and 4.0% in the scenario with a further 20% vector control effectiveness. The total number of infections in this vaccination scenario dropped, ranging from 173,981 to 256,486 and 71,077 to 136,810, respectively (Table 4 and Figure 2).

**Figure 3:**
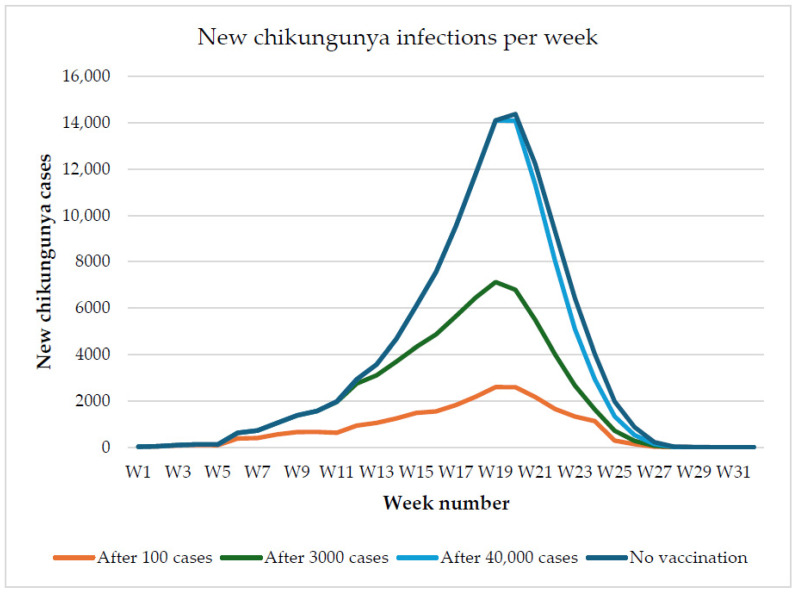
Epidemic curve showing the new weekly chikungunya infections for the median run out of the seven selected simulations. The colors represent the different vaccination scenarios, with vaccination starting after 100 cases are detected (orange), after 3000 cases are detected (green), after 40,000 cases are detected (light blue), and with no vaccination (dark blue).

The epidemic curve of the median run illustrates how variations in the timing of vaccination programs can influence outcomes (Figure 3).

## 4. Discussion

Our model estimated the potential size of the 2024–2025 CHIKV outbreak in La Réunion and evaluated the impact of an emergency vaccination campaign. Assuming 20% additional vector control effectiveness, the outbreak was projected to result in approximately 108,000 infections, compared to around 246,500 infections with only 10% vector control effectiveness. Introducing an emergency vaccination program could prevent up to 75% of these cases. As of mid-August 2025, surveillance data reported 54,545 infections [22], aligning with the 10% vector control scenario. Without vaccine intervention, the current trajectory suggests a total outbreak size of roughly 200,000 infections. Additionally, our model highlights that the window of effective intervention closes rapidly: vaccination initiated when 100 cases are detected could prevent three-quarters of infections, when delaying until 40,000 cases limits prevention to about one-tenth. These findings underscore the importance of early detection, rapid start of vaccination campaigns, and real-time surveillance integration into outbreak response plans.

Beyond vaccination, strengthening vector control measures can be an efficient complementary way to reduce the impact of outbreaks. Enhanced vector control alone could halve the outbreak size, as shown by our study. However, without the implementation of emergency vaccination, the outbreak could still generate over 100,000 infections. Thus, vaccination and vector control should be viewed as mutually reinforcing interventions rather than substitutes [13].

By late June 2025, CHIKV infection had caused 27 deaths and 577 hospitalizations lasting over 24 h. Beyond protecting individuals and helping contain the outbreak, vaccination also alleviates pressure on the healthcare system by reducing hospital bed occupancy during severe outbreaks, considering a symptomatic rate of 75% and chronicity rate of 44% [23]. Additionally, it can substantially lower the economic burden by reducing productivity losses and informal care costs associated with CHIKV infection [24]. Earlier initiation of vaccination maximizes the number of cases that can be averted. While even low vaccination coverage provides value, increased coverage results in a proportionally larger reduction in cases. For future outbreaks, if resources allow, offering vaccination to the entire population and aiming for high coverage could further reduce the impact of emerging chikungunya outbreaks.

At the time of writing, approximately 40,000 IXCHIQ^®^ doses had been delivered as part of a vaccination strategy implemented by local health authorities. The campaign focused on adults aged 65 years and older and those with pre-existing comorbidities, as these groups were considered at higher risk of developing severe disease outcomes. This focus was reflected in our modeling assumptions, which applied vaccination coverage only to these priority groups. If comparable coverage levels were extended across the broader population, our model suggests that vaccination would yield similar overall reductions in infections.

Although a vaccination campaign had been implemented, observed outcomes differ from our projections, likely due to timing. The real-world rollout began on 8 April—around the epidemic peak—whereas our model assumed vaccination initiation at an earlier stage. This timing difference likely explains the discrepancy between the observed and modeled outcomes and underscores a key finding of our analysis: the timing of vaccine deployment critically determines its effectiveness in mitigating an outbreak. Anticipating outbreaks and implementing vaccination campaigns at an early stage are therefore essential to maximize public health impact.

Our findings highlight the need for authorities to enhance preparedness for future outbreaks, particularly in regions highly vulnerable to mosquito-borne diseases. Even in locations that have experienced large outbreaks in the past, the protective effect of natural immunity diminishes over time, leaving these areas vulnerable to new outbreaks. The recurrence of CHIKV has been observed in Rio de Janeiro, Brazil [25]; southern Thailand [26]; Asunción, Paraguay [27]; and currently La Réunion. The case of La Réunion shows that even when a significant proportion of the population has natural immunity, a large-scale outbreak can still occur [13].

Beyond the particulars of La Réunion, our results illustrate a general lesson: real-time, calibrated transmission models can convert uncertain surveillance snapshots into concrete response timelines. In settings where *Aedes* mosquitoes are established, public health authorities can input their own early-outbreak data into the same framework to stress-test contingency plans—identifying when vaccination stockpiles should be mobilized, how aggressively vector control must be scaled, and what caseloads are at stake if responses are delayed.

Our model has several limitations. The timing of the outbreak peak was based on expert opinion, and the end of the outbreak was estimated using historical data; therefore, the actual trajectory of the outbreak may differ from our predicted course. Additionally, the model assumes a homogenous population without spatial heterogeneity or clustering, meaning infections are evenly distributed rather than concentrated in specific areas. However, given the relatively small size of La Réunion and tropical climate, multiple transmission clusters could develop and effectively cover the entire island [25,28], making the homogenous population assumption a reasonable approximation [25]. Although current vector control measures are accounted for through model calibration, assumptions were necessary regarding potential improvements of these measures, with scenarios modeled at a 10% and 20% increase in effectiveness. However, the actual impact of future interventions by authorities in La Réunion remains uncertain.

During the review phase of this paper, the U.S. Food and Drug Administration (FDA) suspended the license of IXCHIQ^®^ on 22 August 2025 following reports of serious adverse events, mainly among older adults with preexisting comorbidities. While these safety events were not incorporated into our model, their occurrence emphasizes that the rate and severity of vaccine-related adverse reactions depend strongly on who is vaccinated. This has important implications for vaccination strategies and prioritization, as targeting populations at highest exposure risk while avoiding those at increased risk of adverse outcomes may optimize the overall benefit–risk balance. Within the EMA, the Pharmacovigilance Risk Assessment Committee (PRAC) concluded that the benefit–risk profile of IXCHIQ^®^ remains favorable when used in individuals with a meaningful risk of chikungunya virus exposure and after individual benefit-risk assessment. Together, these developments highlight the need for continued post-marketing safety monitoring and context-specific vaccination policies that carefully balance protection against chikungunya with potential vaccine-related risks.

## 5. Conclusions

Timely implementation of an emergency IXCHIQ^®^ vaccination program can substantially reduce CHIKV transmission and support outbreak control in *Aedes*-endemic regions. Based on outbreak scenarios informed by the 2024–2025 La Réunion outbreak, this study highlights the value of emergency vaccination for guiding rapid decision-making and strengthening outbreak response. By showing that targeted, early vaccination has the potential to substantially reduce outbreak size and prevent disease burden, these findings emphasize the importance of preparedness and swift public health action against emerging arboviral threats.

## Figures and Tables

**Table 1:** Overview of the model inputs used in the model.

Parameter	Value	Source
**Human population**		
Human population	896,175	[15]
Natural immunity	0.2	[5]
Human susceptibility to infection	0.375	[16]
Intrinsic incubation rate	0.33	[16]
Recovery rate	0.33	[16]
**Mosquito population**		
Mosquito susceptibility to infection	0.375	[16]
Biting rate	Time-dependent	Calibrated
Female-male ratio	0.5	Assumption
Additional mortality	0.05	Assumption
Female mosquito population threshold	896,175	Depends on VHR and female ratio
Initial mosquito population proportion	0.9	Assumption
**General parameters**		
Vector–host ratio	2	Assumption
Start date outbreak	17 November	
Time step	0.2	
**Vaccination parameters**		
Vaccine efficacy	0.989	[10]
Vaccine coverage rate	0.145	Calculation
Cases before outbreak identification	Varies	Multiple scenarios
Vaccination program duration (days)	30	Assumption
Time until vaccination provides immunity (days)	14	[10,17]
**Counter measures**		
Extra vector control	10% or 20%	Assumption

**Table 2:** Estimation of the number of vaccinations within the emergency targeted vaccination program.

Age Group	Total Population	Natural Immunity	Susceptible Population	Vaccination Coverage Rate	Absolute Number of Vaccinations
0–17	224,822 [20]	0.0%	224,822	0.00%	0
18–64 (no comorb.)	350,021 [20]	32.7%	248,064	0.00%	0
18–64 (comorb.)	180,314 [20]	29.1%	121,437	42.72%	77,026
65+	141,018 [20]	49.3%	71,500	37.68%	53,130

**Table 3:** Model predictions display the total number of infections with an additional 10% or 20% vector control.

Additional Vector Control	Simulation Number						
	1	2	3	4	5	6	7
10%	219,202	271,356	298,861	208,880	268,062	270,808	188,926
20%	96,677	116,711	147,514	87,251	117,587	118,615	72,065

**Table 4:** Predicted outbreak sizes per scenario for both the vector control scenarios (10% and 20%) across the seven runs.

Simulation	No Vaccination		Vaccination After 100 Cases		Vaccination After 3000 Cases		Vaccination After 40,000 Cases	
	10% ^1^	20% ^1^	10%	20%	10%	20%	10%	20%
1	219,202	96,677	52,828	22,190	126,898	59,077	197,882	94,136
2	271,356	116,711	64,146	30,597	158,933	69,163	235,933	111,169
3	298,861	147,514	90,726	43,718	178,032	88,059	256,486	136,810
4	208,880	87,251	57,653	28,012	121,929	53,532	190,184	85,285
5	268,062	117,587	59,406	27,111	156,888	70,147	234,245	111,984
6	270,808	118,615	63,464	25,741	159,895	71,161	235,329	112,701
7	188,926	72,065	44,018	15,357	110,575	44,680	173,981	71,077
Average outbreak size	246,585	108,060	61,749	27,532	144,736	65,117	217,720	103,309
Average reduction in cases	0	0	75.1%	74.7%	41.4%	39.6%	11.4%	4.0%

^1^ vector control scenarios.

## Data Availability

All data generated or analyzed during this study are included in this published article.

## Abbreviations

The following abbreviations are used in this manuscript:

CHIKV	Chikungunya virus
EMA	European Medicines Agency
FDA	Food and Drug Administration
HAS	Haute Autorité de Santé
ODE	Ordinary Differential Equation
PRAC	Pharmacovigilance Risk Assessment Committee
SEIR-SEI	Susceptible, Exposed, Infected, Recovered-Susceptible, Exposed, Infected

## References

[1] Gérardin P., Guernier V., Perrau J., Fianu A., Le Roux K., Grivard P., Michault A., De Lamballerie X., Flahault A., Favier F. (2008). Estimating Chikungunya prevalence in La Reunion Island outbreak by serosurveys: Two methods for two critical times of the epidemic. BMC Infect. Dis..

[2] Guillot X., Ribera A., Gasque P. (2020). Chikungunya-Induced Arthritis in Reunion Island: A Long-Term Observational Follow-Up Study Showing Frequently Persistent Joint Symptoms, Some Cases of Persistent Chikungunya Immunoglobulin M Positivity, and No Anticyclic Citrullinated Peptide Seroconversion After 13 Years. J. Infect. Dis..

[3] Josseran L., Paquet C., Zehgnoun A., Caillere N., Tertre A.L., Solet J.-L., Ledrans M. (2006). Chikungunya Disease Outbreak Reunion Island. Emerg. Infect. Dis..

[4] Soumahoro M.-K., Boelle P.-Y., Gaüzere B.-A., Atsou K., Pelat C., Lambert B., La Ruche G., Gastellu-Etchegorry M., Renault P., Sarazin M. (2011). The Chikungunya epidemic on La Reunion Island in 2005–2006: A cost-of-illness study. PLoS Neglected Trop. Dis..

[5] Bardin P., Rakotondrainipiana M., Rouveix E., D’Herbe F., Lasserre A. (2025). Utilisation du Vaccin IXCHIQ dans le Contexte Épidémique de Chikungunya dans les Territoires de La Réunion et de Mayotte.

[6] European Centre for Disease Prevention and Control (2024). Communicable Disease Threats Report, Week 35 24–30 August 2024. https://www.ecdc.europa.eu/en/publications-data/communicable-disease-threats-report-24-30-august-2024-week-35.

[7] European Centre for Disease Prevention and Control (2025). Communicable Disease Threats Report, Week 9, 22–28 February 2025. https://www.ecdc.europa.eu/en/publications-data/communicable-disease-threats-report-22-28-february-2025-week-9.

[8] European Medicines Agency (2024). First Vaccine to Protect Adults from Chikungunya. https://www.ema.europa.eu/en/news/first-vaccine-protect-adults-chikungunya.

[9] European Medicines Agency (2025). New Chikungunya Vaccine for Adolescents from 12 and Adults. https://www.ema.europa.eu/en/news/new-chikungunya-vaccine-adolescents-12-adults.

[10] Schneider M., Narciso-Abraham M., Hadl S., McMahon R., Toepfer S., Fuchs U., Hochreiter R., Bitzer A., Kosulin K., Larcher-Senn J. (2023). Safety and immunogenicity of a single-shot live-attenuated chikungunya vaccine: A double-blind, multicentre, randomised, placebo-controlled, phase 3 trial. Lancet.

[11] Richardson J.S., Anderson D.M., Mendy J., Tindale L.C., Muhammad S., Loreth T., Tredo S.R., Warfield K.L., Ramanathan R., Caso J.T. (2025). Chikungunya virus virus-like particle vaccine safety and immunogenicity in adolescents and adults in the USA: A phase 3, randomised, double-blind, placebo-controlled trial. Lancet.

[12] Sloof A.C., Boer M., Vondeling G.T., de Roo A.M., Jaramillo J.C., Postma M.J. (2024). Strategic vaccination responses to Chikungunya outbreaks in Rome: Insights from a dynamic transmission model. PLoS Neglected Trop. Dis..

[13] Christofferson R.C., Mores C.N. (2015). A role for vector control in dengue vaccine programs. Vaccine.

[14] Jourdain F., de Valk H., Noël H., Paty M.-C., L’Ambert G., Franke F., Mouly D., Desenclos J.-C., Roche B. (2022). Estimating chikungunya virus transmission parameters and vector control effectiveness highlights key factors to mitigate arboviral disease outbreaks. PLoS Neglected Trop. Dis..

[15] World Population Review (2025). Reunion Population. https://worldpopulationreview.com/countries/reunion.

[16] Dumont Y., Chiroleu F. (2010). Vector control for the Chikungunya disease. Math. Biosci. Eng..

[17] Chen L.H., Fritzer A., Hochreiter R., Dubischar K., Meyer S. (2024). From bench to clinic: The development of VLA1553/IXCHIQ, a live-attenuated chikungunya vaccine. J. Travel Med..

[18] Kakarla S.G., Mopuri R., Mutheneni S.R., Bhimala K.R., Kumaraswamy S., Kadiri M.R., Gouda K.C., Upadhyayula S.M. (2019). Temperature dependent transmission potential model for chikungunya in India. Sci. Total Environ..

[19] Yang H., Macoris M., Galvani K., Andrighetti M., Wanderley D. (2009). Assessing the effects of temperature on the population of Aedes aegypti, the vector of dengue. Epidemiol. Infect..

[20] Insee (2025). Estimation de Population au 1er Janvier, par Région, Sexe et âge Quinquennal. https://www.insee.fr/fr/statistiques/8331297.

[21] Wressnigg N., Hochreiter R., Zoihsl O., Fritzer A., Bézay N., Klingler A., Lingnau K., Schneider M., Lundberg U., Meinke A. (2020). Single-shot live-attenuated chikungunya vaccine in healthy adults: A phase 1, randomised controlled trial. Lancet Infect. Dis..

[22] European Centre for Disease Prevention and Control (2025). Communicable Disease Threats Report; Week 35 23–29 August 2025. https://www.ecdc.europa.eu/en/publications-data/communicable-disease-threats-report-23-29-august-2025-week-35.

[23] Rama K., de Roo A.M., Louwsma T., Hofstra H.S., Gurgel do Amaral G.S., Vondeling G.T., Postma M.J., Freriks R.D. (2024). Clinical outcomes of chikungunya: A systematic literature review and meta-analysis. PLoS Neglected Trop. Dis..

[24] de Roo A.M., Vondeling G.T., Boer M., Murray K., Postma M.J. (2024). The global health and economic burden of chikungunya from 2011 to 2020: A model-driven analysis on the impact of an emerging vector-borne disease. BMJ Glob Health.

[25] Dalvi A., Braga J. (2019). Spatial diffusion of the 2015–2016 Zika, dengue and chikungunya epidemics in Rio de Janeiro Municipality, Brazil. Epidemiol. Infect..

[26] Ammatawiyanon L., Tongkumchum P., McNeil D., Lim A. (2023). Statistical modeling for identifying chikungunya high-risk areas of two large-scale outbreaks in Thailand’s southernmost provinces. Sci. Rep..

[27] Torales M. (2023). Notes from the field: Chikungunya outbreak—Paraguay, 2022–2023. Morb. Mortal. Wkly. Rep..

[28] Freitas L.P., Carabali M., Yuan M., Jaramillo-Ramirez G.I., Balaguera C.G., Restrepo B.N., Zinszer K. (2022). Spatio-temporal clusters and patterns of spread of dengue, chikungunya, and Zika in Colombia. PLoS Neglected Trop. Dis..

